# Impact of continuing or quitting smoking on episodic cluster headache: a pilot survey

**DOI:** 10.1186/1129-2377-14-48

**Published:** 2013-06-06

**Authors:** Anna Ferrari, Maurizio Zappaterra, Federica Righi, Michela Ciccarese, Ilaria Tiraferri, Luigi Alberto Pini, Simona Guerzoni, Maria Michela Cainazzo

**Affiliations:** 1 Headache and Drug Abuse Inter-Department Research Centre, Division of Toxicology and Clinical Pharmacology, University of Modena and Reggio Emilia- Policlinico, Largo del Pozzo, 71-41100 Modena, Italy; 2 School of Medical Toxicology, University of Modena and Reggio Emilia, Modena, Italy; 3 School of Pharmacology, University of Modena and Reggio Emilia, Modena, Italy

**Keywords:** Cluster headache, Smoking, Cigarette, Nicotine, Pain

## Abstract

**Background:**

The majority of patients suffering from cluster headache (CH) are smokers and it has been suggested that smoking may trigger the development of CH. The aim of this pilot survey was to describe: 1. the differences between current, former, and never smokers CH patients; 2. if smoking changed during an active cluster period; 3. if CH changed after quitting.

**Methods:**

All outpatients with episodic CH according to the criteria of ICHD-II who were consecutively seen for the first time from October 2010 to April 2012 at a headache centre were interviewed by phone using a specifically prepared questionnaire. Statistical differences between continuous variables were analysed by the Student’s *t-*test or the one-way analysis of variance (ANOVA), followed by Newman-Keuls post-hoc testing. Comparisons between percentages were made using the Chi-square test or Fisher’s exact test. All data were expressed as the mean ± standard deviation (SD).

**Results:**

Among a total of 200 patients surveyed (172 males, 28 females; mean age ± SD: 48.41 ± 12 years) there were 60%, 21%, and 19% of current, former, and never smokers, respectively. Current smokers reported longer active periods (12.38 ± 10 weeks) and a higher maximum number of attacks per day (3.38 ± 1) compared to never smoker CH patients (5.68 ± 4 weeks, P <0.05 and 2.47 ± 1, P <0.05, respectively). During the active period most of the patients stated to decrease (45.7%) or not to change (45.7%) the number of cigarettes smoked. Among those who decreased smoking, most (83.8%) reported that they had less desire to smoke. After quitting, the majority of former smokers stated that their headache had not changed.

**Conclusions:**

Patients with episodic CH who are also smokers appear to have a more severe form of the disorder. However, it is unlikely that between CH and smoking there is a causal relationship, as CH patients rarely improve quitting smoking.

## Background

The pathophysiology of cluster headache (CH), a very severe form of primary headache [1], is not yet fully known [2]. It is assumed that the activation of the posterior hypothalamus and the trigeminovascular system is involved [3] and probably genetic [4] and environmental factors are also important [5]. Among the latter, tobacco usage has always been consistently identified as associated with CH [6-8]. Indeed, most of CH patients, up to 90% of males and about 70% of females, are smokers [9]. This high prevalence of smokers, greater than in the general population [7], supports the possibility that smoking may trigger the development of CH [10-12]. In favour of a causal relationship between smoking and CH, it has been observed that in most cases the onset of the smoking habitus precedes the beginning of CH [6,8,13-15] and that the prolonged exposure to second hand smoke during childhood may trigger CH even in non-smokers [12,16]. Moreover, CH develops earlier in people living with smoking parents [12]. The exposure to secondary smoke of parents could have an even more important role in causing CH in women, among which the prevalence of smoking is lower than in men [16]. The number of women with CH is increasing and one of the possible causes of this trend could be the rising prevalence of smoking among women [9,14,16,17]. Finally, just the decline of the epidemic of smoking in Minnesota was considered a factor that has helped reduce the incidence of CH from 1979 to 1990 [15].

The significance of the association with smoking for the pathophysiology of CH remains unclear. Nicotine and/or other toxic agents present in tobacco-based products may cause CH acting directly on the hypothalamus [12]. Smoking could trigger the activation of the trigeminal autonomic reflex at the level of the brainstem, causing precisely the cranial autonomic symptoms typical of CH [18]. If CH was triggered and/or worsened by smoking, then the fight against cigarette smoking and the interventions to help patients quit smoking would become priority to reduce respectively the incidence of CH and the patients’ suffering.

One of the strongest proofs to establish the existence of a causal link between a substance and an event is its decrease/disappearance by discontinuing the intake of the substance. In fact, several diseases caused by smoking improve quickly if the patient stops smoking. However, the impact of continuing or quitting smoking on the clinical presentation and the time course of CH has not been much studied. Thus, the purpose of our pilot survey was to describe, by means of a questionnaire administered by telephone, the impact of the smoking status on episodic CH patients consecutively seen at a headache centre, exploring: 1. the differences between current, former, and never smokers; 2. if the mode of smoking changed in current smokers during an active cluster period 3. if CH had changed in former smokers after they had stopped smoking.

## Methods

### Patients

We enrolled all female and male outpatients, of all ages, consecutively examined at the Headache Centre of the University Hospital of Modena at a first visit from October 2010 to April 2012. We only included patients who had been diagnosed episodic cluster headache according to the criteria of the International Classification of Headache Disorders, 2nd edition (ICHD-II) [1]. A total of 207 patients were found and they represented 7.2% of the 2881 patients globally examined for the first visit in the period of the study. All patients were asked, during the visit, their written informed consent to take part in the survey; none of them refused to give it. However, it was impossible to contact 7 patients. Our sample consisted therefore of 200 episodic cluster headache patients. The study was approved by the Ethical Committee of Modena and it was conducted in compliance with the Declaration of Helsinki, latest version.

### Questionnaire and procedures

A questionnaire was prepared for the study. It included a demographic section and a section investigating the characteristic of CH (age of onset, frequency and length of active periods, and maximum number of attacks/day in the active period) and whether patients were current, former or never smokers. Those who declared themselves current or former smokers were asked to specify the age of onset of smoking, the number of cigarettes smoked at the beginning and presently, and if in the active period of CH their mode of smoking increased, decreased or was unchanged and why. Former smokers were asked how long before they had quit smoking and if this had increased, decreased or unchanged the characteristics of the headache (frequency and duration of the active periods, maximum number of attacks per day, length and intensity of the single attack).

Before being administered, the questionnaire had been tested among the patients seen at the outpatients’ ward of the Headache Centre in July, August, and September 2010. The questionnaire was administered during a phone interview by a trained postgraduate medical doctor who had never examined the patients before.

### Data analysis

The data collected by the questionnaire were inserted into a specially prepared data-base. A descriptive analysis of all collected parameters was made. In order to compare the three groups defining smoking status (current, former, and never smokers) in terms of continuous variables (such as age, age of disease onset, length of CH, length of active periods, and maximum number of attacks/day), means and standard deviations (SD) for each group were calculated and the one-way analysis of variance (ANOVA) was conducted, followed by Newman-Keuls post-hoc testing. In terms of categorical variables (such as sex, level of education, occupation status, marital status, and number of active periods), counts and percentages for each group were obtained and a Chi-square test or a Fisher’s exact test was conducted. For multiple comparison, a Bonferroni correction was carried out and a *P* value < 0.025 was considered significant. Moreover, a comparison was made between female and male episodic CH patients.

In order to compare current and former smokers in terms of continuous variables (such as age of onset of smoking, length of smoking, number of cigarettes/day smoked at the onset of CH, and number of cigarettes/day smoked at the time of completing the questionnaire), means ± SD were calculated between groups and the Student’s t-test was carried out. In terms of categorical variables (such as patients who start smoking before the onset of CH, changes in the smoking status – defined as increased, decreased or unchanged - during the active period), counts and percentages for each group were obtained and a Chi-square test or a Fisher’s exact test was conducted. A *P-*value < 0.05 was considered significant.

## Results

Current smokers represented 60% of the sample, former smokers 21%, and never smokers 19%. Males were the majority of the sample. Overall, the M/F ratio was 6.14:1; the ratio was not similar among the three groups (*P* < 0.05): it did not statistically differ between current smokers and never smokers (respectively 5.66:1 and 2.8:1), but it was statistically different between former smokers and the other two groups, because there were no women among former smokers.

The mean age (Table 1) was similar among current, former, and never smokers. Women were significantly older (54.21 ± 8.15 years) than men (47.46 ± 11.9 years) (*P* < 0.05). More than half of the sample (55%) had at least a diploma. Former and never smokers were generally more educated than current smokers (respectively 66.8% and 63.1% versus 48.4% with at least a diploma). The percentage of graduates was significantly higher among women (35.7%, n = 10/28) than among men (15.1%, n = 26/172, *P* < 0.05). The majority of the sample (80%) worked, with no differences between females (n = 20/28, 71.4%) and males (n = 140/172, 81.4%). Sixty percent of the patients were living with a partner/spouse, with no difference between females (n = 12/28, 42.8%) and males (n = 106/172, 61.6%).

**Table 1:** Demographic data

**Variable**	**Total (n = 200)**		**Current smokers (n = 120)**		**Former smokers (n = 42)**		**Never smokers (n = 38)**	
Mean age **±** SD (years)	48.41 ± 12		47.91 ± 11		50.95 ± 12		47.16 ± 16	
Range	25 - 85		25 - 85		32 - 78		30 - 81	
	*N.*	*%*	*N.*	*%*	*N.*	*%*	*N.*	*%*
Females	28	14	18^a^	15	0	0	10	26.3
Males	172	86	102	85	42^b^	100	28	73.7
*Education*								
Primary school	20	10	8	6.6	2	4.7	10^c, d^	26.3
Middle school	70	35	54^e^	45	12	28.5	4	10.6
High school	74	37	40^a^	33.4	24	57.3	10^d^	26.3
University degree	36	18	18	15	4	9.5	14^d, e^	36.8
*Occupation*								
Employed	160	80	94	78.3	36	85.7	30	78.9
Housewife	2	1	2	1.7	0	0	0	0
Retired person	28	14	14	11.7	6	14.3	8	21.1
Unemployed	10	5	10	8.3	0	0	0	0
*Marital status*								
Living with a partner	120	60	70^a^	58.3	14	33.3	22	57.9
Living alone	80	40	50	41.7	28	66.7	16	42.1

^a ^Current *vs*. former smokers: *P *< 0.025; ^b^ Former *vs. *never smokers: *P *< 0.001; ^c ^Never *vs.* current smokers: *P *< 0.025; ^d ^Never *vs. *former smokers: *P *< 0.025; ^e ^Current vs. never smokers: *P *< 0.025, Chi-square test with Bonferroni correction.

On average, CH began around 30 years (Table 2), with no differences by gender (age of onset of CH, females: 32 ± 18.5, males: 29.45 ± 12.6). The average length of CH was around 18 years and there were no differences between females (22.21 ± 12, range 7–35 years) and males (18.3 ± 10.8, range 1–58 years). Current smokers reported longer active periods and a higher maximum number of attacks than never smokers (*P* < 0.05, ANOVA followed by Newman-Keuls post-hoc testing). The length of the active period was similar between females (12.36 ± 11.07 weeks) and males (12.03 ± 10.8 weeks) and also the maximum number of attacks per day did not vary by gender (females: 3.14 ± 1.9; males: 3.26 ± 2.3). About a third of the sample, with no differences between males and females, had one or more active periods each year; another third had periods of remission exceeding one year; in the remaining, the frequency of active periods was erratic.

**Table 2:** Characteristics of episodic cluster headache in current, former, and never smokers patients

**Variable**	**Total (n = 200)**		**Current smokers (n = 120)**		**Former smokers (n = 42)**		**Never smokers (n = 38)**	
	***Mean *± *SD***	***Range***	***Mean *± *SD***	***Range***	***Mean *± *SD***	***Range***	***Mean *± *SD***	***Range***
Age of onset (years)	29.81± 13	11 - 74	29.63 ± 12	11- 65	28.66 ± 13	15 - 74	31.63 ± 17	16 - 72
Length of CH (years)	18.6 ± 11	1 - 49	18.28 ± 9	2 - 40	22.28 ± 12^a^	4 - 58	15.52 ± 13	1 - 49
Length of active period (weeks)	10.24 ± 8	1 - 40	12.38 ± 10^b^	2 - 40	8.24 ± 7	1 - 40	5.68 ± 4	1 - 20
Maximum number of attacks/day	3.14 ± 1	1 - 8	3.38 ± 1^c^	1 - 8	3.04 ± 2	1 - 8	2.47 ± 1	1 - 6
*N. of active period*	*N.*	*%*	*N.*	*%*	*N.*	*%*	*N.*	*%*
1/year	38	19	26	22	0	0	12	31
>1/year	27	13.5	17	14	4	10	6	16
<1/years	72	36	43	36	17	40	12	32
Erratic	63	31.5	34	28	21^d^	50	8	21

^a ^Former *vs. *current and *vs*. never smokers: *P* < 0.05; ^b ^Current *vs *formers *vs. *and *vs.* never smokers: *P *< 0.05; ^c ^Current *vs*. never smokers: *P *< 0.05, ANOVA followed by Newman-Keuls post-hoc testing; ^d^ Former *vs. *current and *vs*. never smokers: *P *< 0.025, Chi-square test with Bonferroni correction.

Smokers (current and former) (Table 3) had started smoking on average around 17 years and had continued for at least 26 years. There were no differences by gender in terms of age of smoking onset (females: 19.33 ± 7 years, males: 16.76 ± 2.7 years) and in the duration of the smoking habit (females: 32.55 ± 10.3 years, males: 31.54 ± 11.3 years). The vast majority of the patients had started smoking before the beginning of CH (92.6%), with no difference between females (n = 18/18, 100%) and males (n = 132/150, 91.7%). At the onset of CH, patients smoked an average of 17 cigarettes per day; males smoked more intensively (18.39 ± 8.5 cigarettes/day) than females (13.22 ± 6.8 cigarettes/day) (*P* < 0.05). At the time of the interview, the number of cigarettes/day that current smokers claimed smoking had not significantly increased and was similar between females (15.11 ± 8.2 cigarettes/day) and males (17.33 ± 8.4 cigarettes/day). Former smokers, all males, had stopped smoking 8.4 ± 6.8 years before on average (range 1–35 years); in particular, 69% (n = 29/42) of the sample had quit it over 5 years before. During the active period of CH, most patients stated whether to decrease or not to change the number of cigarettes smoked; only a small portion claimed to increase it (8.6%). This pattern was similar between females (decreased: n = 12/18, 66.7%; did not change: n = 6/18, 33.3%; increased: n = 0/18) and males (decreased: n = 63/144, 43.75; did not change: n = 67/144, 46.52%; increased: n = 14/144, 9.8%). Those who did not change the number of smoked cigarettes declared that smoking had no influence on their CH. Among those who decreased smoking, the majority (n = 62/74, 83.8%) reported that they had less desire to smoke and the others (n = 12/74, 16.2%) said that smoking made them feel worse, by increasing the intensity of pain. The few patients who increased smoking said that it made their pain more bearable, relieving agitation. There were no differences by gender in the distribution of the reasons to reduce, not to vary or increase the number of cigarettes smoked during the active period of CH.

**Table 3:** Characteristics of smoking status of episodic cluster headache patients who were current and former smokers

	**Total (n = 162)**		**Current smokers (n = 120)**		**Former smokers (n = 42)**	
	***Mean *± *SD***	***Range***	***Mean *± *SD***	***Range***	***Mean *± *SD***	***Range***
Age of onset of smoking (years)	17.04 ± 3	12 - 38	17.16 ± 3	12 - 38	16.71 ± 2	13 - 22
Length of smoking (years)	29.56 ± 11	2 - 55	30.75 ± 10^a^	11 - 49	26.19 ± 13	2 - 55
N. of cigarettes/day smoked at the onset of cluster headache	17.74 ± 8	1 - 60	17.50 ± 9	1 - 60	17.77 ± 6	6 - 30
N. of cigarettes/day smoked at the time of completing the questionnaire	16.98 ± 8	1 - 35	16.98 ± 8	1 - 35	0	0
	*N*	*%*	*N*	*%*	*N*	*%*
Patients who start smoking before the onset of cluster headache	150	92.6	111	92.5	39	92.85
*Changes in smoking during the active period*						
Increased	14^b^	8.6	12^b^	10	2^b^	5
Decreased	74	45.7	58	48.3	16	40
Unchanged	74	45.7	50	41.7	24	55

^a ^Current *vs. *former smokers: *P *< 0.05, Student’s *t*-test for unpaired data; ^b ^Increased *vs. *decreased and *vs.*unchanged: *P *< 0.0001, Chi-square test with Bonferroni correction.

**Figure 1:**
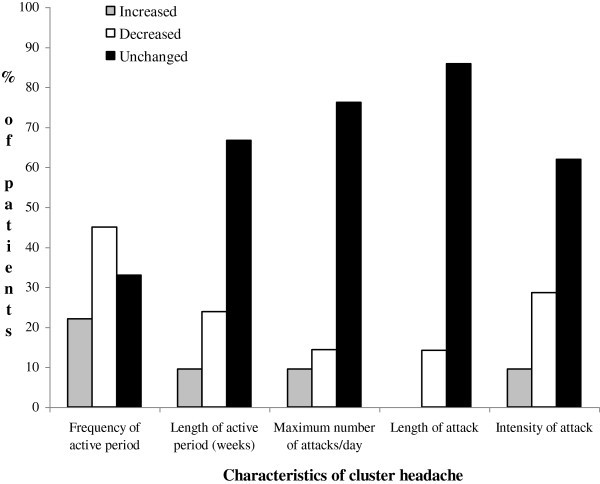
Changes in the characteristics of episodic cluster headache that were reported by 42 former smokers after they had quit smoking.

After they had stopped smoking (Figure 1), most former smokers reported that their headache had not changed. In particular, the patients who did not report any change in the length of the active phase (n = 28/42, 66.7%), the maximum number of attacks/day (n = 32/42, 76.2%), the intensity (n = 26/42, 61.9), and the length of the single attack (n = 36/42, 85.8%) were more than those who stated a reduction of these variables (length of the active period: n = 10/42, 23.8%; number of attacks/day: n = 6/42, 14.3%; intensity: n = 12/42, 28.6%; length of the single attack: n = 6/42, 14.3%). A minority of patients reported an increase in the length of the active period (n = 4/42, 9.5%), the number of attacks/day (n = 4/42, 9.5%), and the intensity of the attack (n = 4/42, 9.5%). Nine out of 42 (22%) declared an increase, 19/42 (45%) a reduction, and 14/42 (33%) no change as far as the frequency of active periods is concerned.

## Discussion

The results of our survey indicated that the smoking status was associated with a more severe phenotype of CH than that of patients who had never smoked. In fact, current smokers reported longer active periods and a higher maximum number of attacks per day compared to never smoker CH patients (*P* < 0.05). Most studies in pain populations find that smoking is related to more pain and greater pain intensity compared to people who never smoked [19,20]. The reasons for this relationship are not clear. Experimental studies in human models of pain suggest that nicotine has analgesic properties [21,22]. However, epidemiological and clinical data show that smoking is a risk factor for chronic pain [23,24]. The finding from current study that former smokers were indistinguishable from never smokers with respect to the length of active periods and the maximum number of attacks per day further strengthens the many reasons why quitting smoking may be beneficial. Given the high smoking rate among CH patients, providing smoking cessation assistance would likely be a valuable addition to treatment.

Patients with pain who are smokers use smoking to manage pain-related emotional distress and as a distracter from pain [25]. Up to 93% of patients with CH report a sense of agitation and restlessness during the attack [7,26,27]. Despite this, most patients said that, during active periods, they did not change smoking or smoked less because their desire to smoke decreased (83.8%) or because smoking worsened pain (16.2%). Maybe this aversive effect was partly caused by the symptoms of cranial autonomic activation, such as lacrimation, nasal congestion, and/or rhinorrhea, which are present in the vast majority of patients [11,27], and may have made quite unpleasant to smoke. Other symptoms, often present during the attack, such as discomfort for strong smells, nausea and vomiting [7,27], may have contributed to reduce smoking or prevented from smoking more to relieve restleness. The behaviour of smokers during active periods of CH has not been much studied. In part, our results confirm what has been reported in a study involving 49 patients with CH: namely, while most patients reduce alcohol intake in active phase, they do not change tobacco use [6]. A recent survey shows that only 27% of patients use cigarettes to relax during or after an attack [8].

It is very unlikely that the association between smoking and CH is a causal relationship. Differently from other diseases caused by smoking, CH had not improved in most patients who had stopped smoking years before (an average of about 8 years). In line with these results, an Internet-based survey finds that only 3% of CH patients have an improvement after quitting smoking [28]. There is probably no correlation between smoking cessation and changes in the course of CH [15].

The present study does not allow clarifying the significance of the association between smoking and CH. It has been suggested that in CH patients the smoking habitus could represent a personality/style-related characteristic [6,29,30]. Differently, it has been speculated that there is a genetic link that predisposes people with CH to nicotine addiction [7]. Moreover, CH patients might be biologically more vulnerable to compulsive pursuit of legal and illegal substances for a partial overlap/sharing of pathways which mediate addiction and are involved in the pathophysiology of CH. Indeed, changes in the prefrontal cortex have recently been found in CH patients [31,32], but dysfunction in prefrontal cortex is also involved in drug addiction [33]. Orexin neuropeptide systems have been suggested to have an important role in both CH pathogenesis [34] and the regulation of the reinforcing properties of most major drugs of abuse [35], including nicotine [36]. A support of this hypothesis is that patients suffering from CH have a greater prevalence of the use of illicit drugs compared to healthy controls [37].

The results of our investigation are similar to those in literature [6,11,13,14,27,38,39] with regard to the prevalence of males and smokers, the long duration of the disorder, and the data indicating that the majority of patients (over 90%) began to smoke before the beginning of CH. In Italy there has been a decline of smoking prevalence for both sexes since 1990 [40]. In 2002, 27.6% of the population reported to be current smoker, while in 2012 this percentage had declined to 20.8% (females: 17.2%, males: 24.6%) of the population [41]. Perhaps this trend explains why the percentage of current smokers (60%) in our study was lower and those of former (21%) and never smokers (19%) higher than in other studies: a survey in Germany between 2002 and 2004 found that current, former, and never smokers among CH patients were respectively 65.9%, 14.2%, and 19.9% [7]. In the same country, there were 68% of current smokers, 19% of former, and 13% of never smokers between 2009 and 2010 [39]. In another survey, carried out in France in 2005, current smokers were 68%, former smokers 19%, and never smokers 12% [11].

Overall, episodic CH seems to appear in a similar way between females and males [42]. There were no striking differences by gender in the variables that we investigated. As reported by a survey in the United States [42], there were significantly more women than men among patients with CH who had never smoked (*P* < 0.005, Fisher's exact test). Differently from this study [42], we found that the probability of never having smoked before the start of CH was similar between females and males, not higher among females. This discrepancy may be due to having conducted the survey among patients of a specialist centre and to the limited number of women in our sample. Male/female ratio is higher in clinical population than in the general population [43]. In the total sample, the M/F ratio was similar to that detected in another Italian study that involved clinical cases series [43].

Our research has some limits. It was carried out among a consecutive series of episodic CH patients seen for a first visit at a specialist centre and the findings cannot be generalised to other patients and settings. In particular, we cannot exclude that there are patients whose cluster headache has completely resolved by quitting smoking and therefore they do not need to seek medical attention. However, if quitting smoking had a decisive impact to improve cluster headache, this effect should apply to all patients with this disorder. The patients who participated in our pilot survey had the typical characteristics of cluster headache and they had received a precise diagnosis of episodic CH according to the ICHD-II classification [1] by a specialist of the headache centre. We assessed the impact of smoking on CH asking the patients’ opinion, which is subject to potential recall bias. The true natural history of cluster headache is very difficult to determine. A prospective study in patients with chronic cluster headache could possibly provide relatively more objective data. Nevertheless, nobody can say better than the patient if his/her health has or has not improved after a change in lifestyle with respect to the prior condition. Moreover, we cannot exclude that some former smokers continued to be exposed to second-hand smoke. Considering that the majority of patients worked (in Italy it is forbidden to smoke in public and private workplaces) and nearly 67% lived alone, it is unlikely that this second-hand smoke exposure was relevant. Finally, there are no data showing that CH patients’ opinion is unreliable. On the contrary, they have been considered to report information accurately [7].

To the best of our knowledge, this is the first study that has specifically described whether and how smoking impacts on CH comparing former to current and never smoker patients.

## Conclusions

The results of our pilot survey show that CH patients who are also smokers have a more serious disorder. These results provide one more reason to advise patients to quit smoking. However, it is important not to blame the patients, since it is unlikely that their CH has been caused by smoking. Indeed, according to most patients’ judgment, quitting smoking has hardly ever any impact on the clinical course of CH.

## Competing interests

The authors declare that that they have no competing interests.

## Authors’ contributions

AF partecipated in designing the survey and wrote the first version of the manuscript. MZ partecipated in designing the survey and performed the statistical analysis. FR administered the questionnaire. MC, IT, LAP, and SG participated in the design of the survey and in critically revising the manuscript. MMC conceived of the study and participated in its design and coordination. All authors read and approved the final manuscript.
